# Association of Dietary n3 and n6 Fatty Acids Intake with Hypertension: NHANES 2007–2014

**DOI:** 10.3390/nu11061232

**Published:** 2019-05-30

**Authors:** Jiahao Chen, Baoqi Sun, Dongfeng Zhang

**Affiliations:** Department of Epidemiology and Health Statistics, the School of Public Health of Qingdao University, No. 38 Dengzhou Road, Qingdao 266021, China; chenjh1208@126.com (J.C.); SunBaoQi1214@163.com (B.S.)

**Keywords:** hypertension, dietary n3 fatty acid, dietary n6 fatty acid, n6:n3 ratio, dose-response

## Abstract

We conducted this cross-sectional study in the American general population to explore the association of dietary n3 and n6 fatty acids intake and the risk of hypertension. We used data from the National Health and Nutrition Examination Survey (NHANES) 2007–2014 in this study. We obtained dietary n3 and n6 fatty acids data through two 24 h dietary recall interviews and n3, n6 fatty acids intake were adjusted by weight. We defined hypertension as now taking prescribed medicine for hypertension or blood pressure above 130/80 mmHg. We applied binary logistic regression, multinomial logistic regression, and restricted cubic spline to evaluate the associations of dietary n3 and n6 fatty acids intake with hypertension. A total of 18,434 participants were included in this study. In the multivariate-adjusted model 2, the odds ratios (ORs) with 95% confidence interval (CI) of hypertension were 0.58 (0.49–0.68), 0.53 (0.45–0.63), and 0.92 (0.80–1.06) for the highest versus the lowest tertile of dietary n3, n6 fatty acids intake and n6:n3 ratio, respectively. Further excluded participants with hypertension history, the ORs with 95% CI of newly diagnosed hypertension were 0.60 (0.50–0.73), 0.52 (0.43–0.62), and 0.95 (0.79–1.14) for the highest versus lowest tertile of dietary n3, n6 fatty acids intake and n6:n3 ratio, respectively. Dose-response analyses showed that the risk of hypertension was associated with dietary n3 and n6 fatty acids intake. Our study suggested that dietary n3 and n6 fatty acids intake were inversely associated with the risk of hypertension in US adults.

## 1. Introduction

The data from the World Health Organization (WHO) showed that the prevalence of hypertension was approximately 22% globally among adults in 2014, and it was expected to rise to 29.2% in 2025 without intervention [1]. In the past about 20 years, the prevalence of hypertension among U.S adults was about 32% and remained at a high level, and there were no significant changes from 1999 to 2014 [2]. At the same time, the expenditures on the treatment and prevention of hypertension are enormous; as reported, the US spent an estimated $51.2 billion from 2012 to 2013 [3]. Hypertension is one of the most common chronic diseases and a major risk factor for disease burden in the world [4], meanwhile it could increase the risk of stroke, cardiovascular disease, kidney failure, dementia, and Parkinson’s disease and so on [5,6,7,8,9]. In view of the huge disease burden of hypertension, the American College of Cardiology (ACC) and American Heart Association (AHA) adjusted the diagnostic criteria for hypertension to 130/80 mmHg in 2017 [10]. It is essential to pay attention to investigate the modifiable risk factors for hypertension and then take the corresponding measures to prevent and control it.

In recent years, epidemiologic studies were conducted to investigate the association between dietary factors and hypertension, such as fruit and vegetables, whole grain, meat, protein, fiber, copper, zinc, and fatty acids [11,12,13,14,15,16,17,18,19]. Among these dietary factors, some are protective factors, such as fruit and vegetables [11,12,13], whole grains [14], and fiber [15]. There are also some risk factors, such as meat [14], a high-salt diet [14], and so on.

n3 and n6 fatty acids are two important components of polyunsaturated fatty acids, with the first of the double bonds in the cis configuration starting from the third and sixth carbon atom, respectively [20]. Studies found n3 and n6 fatty acids were associated with many diseases, such as cardiovascular diseases [21,22,23,24,25], obesity [26,27], metabolic syndrome [23,27,28,29], and diabetes [30,31,32,33]. Meanwhile, some studies found n3, n6 fatty acids intake could decrease the risk of hypertension [34,35,36] but other studies founded there were no association between n3, n6 fatty acids intake and n6:n3 ratio and the risk of hypertension [37,38,39]. In view of the inconsistency of the above results, no relevant studies had been reported among the American population after the 2017 new hypertension diagnostic criteria [10] was released, and there was a lack of studies that performed a dose-response relationship. Therefore, we conducted this cross-sectional study using new hypertension diagnostic criteria and using data from NHANES 2007–2014 to explore the associations between n3, n6 fatty acids intake and n6:n3 ratio and the risk of hypertension, and to explore the corresponding dose-response relationships.

## 2. Materials and Methods

### 2.1. Data Source and Study Population

The purpose of National Health and Nutrition Examination Survey (NHANES) is to assess the health and nutritional status among the US population and NHANES adopted a stratified multistage probabilistic sampling method to select a representative sample of the civilian non-institutionalized US population. This study was approved by the National Center for Health Statistics Research Ethics Review Board and informed consent was obtained from every participant.

**Figure 1:**
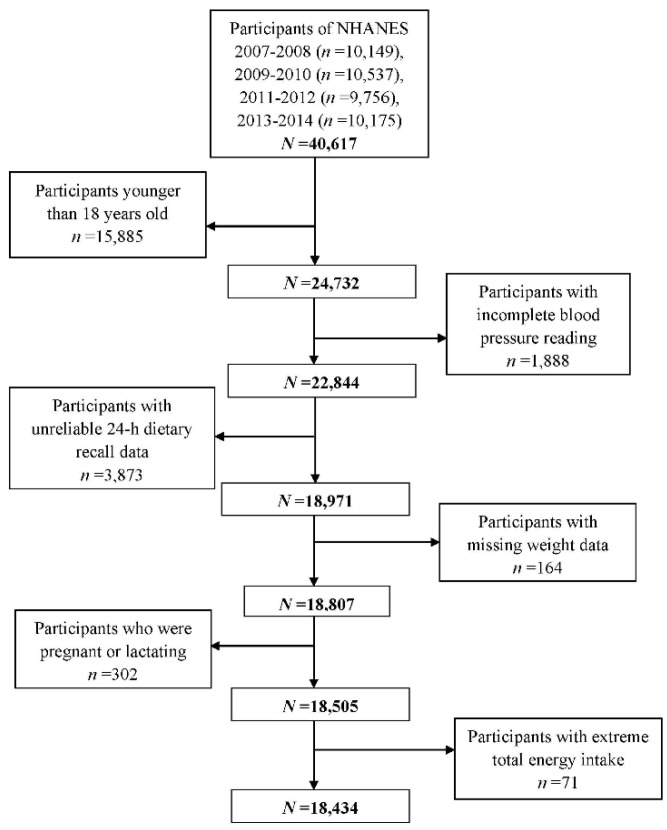
Flow chart of the screening process for the selection of eligible participants.

Publicly available data from NHANES 2007–2008, 2009–2010, 2011–2012, and 2013–2014 were used in this article. In NHANES 2007–2014, there were a total of 40,617 individuals and our analyses were limited to 24,732 individuals aged 18 years and older. Among them, the individuals without complete blood pressure readings (*n* = 1888), with unreliable 24 h recall data (*n* = 3873), and with missing weight data (*n* = 164) were further excluded. Then, we continued to exclude females who were pregnant or lactating (*n* = 302) and the individuals whose total energy intake less than 500 or above 5000 kcal/day for females, and less than 500 or above 8000 kcal/day for males (*n* = 71). In the end, a total of 18,434 participants were included in this cross-sectional study (Figure 1).

### 2.2. Blood Pressure and Definition of Hypertension

Among participants, their blood pressure was measured using a protocol that follows procedures developed by the American Heart Association [40]. Participants rested quietly in a sitting position for 5 min and then their blood pressure was measured three times using a mercury sphygmomanometer by examiners who were certified through a training program. A fourth reading was measured if required and the means of systolic blood pressure (SBP) and diastolic blood pressure (DBP) were calculated.

Hypertension was defined as SBP ≥ 130 mmHg and/or DBP ≥ 80 mmHg or if the participant reported he/she was currently taking medications for hypertension. Newly diagnosed hypertension was defined as SBP ≥ 130 mmHg and/or DBP ≥80 mmHg and without history of hypertension. Stage 1 hypertension was defined as 130 mmHg ≤ SBP < 140 mmHg and/or 80 mmHg ≤ DBP < 90 mmHg, stage 2 hypertension was defined as SBP ≥ 140 mmHg and/or DBP ≥ 90 mmHg. 

### 2.3. Dietary n3 and n6 Fatty Acids Intake

Dietary n3 and n6 fatty acids intakes were obtained from two 24 h dietary recall interviews. The two interviews were collected in-person in the mobile examination center (MEC) and by telephone 3 to 10 days later, respectively. Because linolenic acid includes primarily “alpha-linolenic acid” (n3) and lesser amounts of “gamma-linolenic acid” (n6) [41] and there was not a detailed classification of linolenic acid in NHANES, we categorized linolenic acid into n3 fatty acid. Thus, in our analyses, n3 fatty acid contained linolenic acid (18:3), stearidonic acid (18:4), eicosatetraenoic acid (20:5), clupanodonic acid (22:5), and docosahexaenoic acid (22:6), and n6 fatty acid contained linoleic acid (18:2) and arachidonic acid (20:4). The average daily dietary n3, n6 fatty acids were calculated according to the U.S. Department of Agriculture’s Dietary Research Food and Nutrition Database for Dietary Studies [42] and were adjusted to the body weight. Dietary n3, n6 fatty acids intake and n6:n3 ratio were divided into tertiles.

### 2.4. Covariates

The following demographic characteristics were included to control the potential effects of confounding: Age (18–39 years, 40–59 years, 60–79 years, and ≥80 years), gender (Male and Female), race (Mexican American, Other Hispanic, Non-Hispanic White, Non-Hispanic Black, and Other race), educational level (Below high school, High school, and Above high school), annual household income (<$20,000 and ≥$20,000), work activity (Vigorous activity, Moderate activity, and Other), recreational activity (Vigorous activity, Moderate activity, and Other), smoking (Smoking at least 100 cigarettes in life or not), drinking (Having at least 12 alcohol drinks per year or not), diabetes (Yes/No) and total daily energy intake. Diabetes was defined according to self-reported diabetes history and total daily energy intake was obtained from two 24 h dietary recall interviews and two 24 h dietary supplement recall interviews.

### 2.5. Statistical Analysis

We tested the normality of continuous variables by Kolmogorov-Smirnov normality tests and described normal distributed variables with mean ± standard deviation, non-normal distributed variables with median (interquartile range). We adopted Student’s *t*-tests to compare the mean levels between the hypertension group and the non-hypertension group if the variable was normally distributed, otherwise, the Mann-Whitney *U* test was adopted. And we adopted Chi-square tests to compare the percentages of categorical variables between the hypertension group and the non-hypertension group. Dietary n3, n6 fatty acids intakes and n6:n3 ratio were categorized based on tertiles (tertile 1: <33th percentile, tertile 2: ≥3 to 67th percentile, tertile 3: ≥67th percentile), and tertile 1 was the referent category. We conducted binary logistic regression analyses to examine the association of dietary n3, n6 fatty acids intake and n6:n3 ratio with hypertension. Model 1 adjusted for age and gender. Model 2 adjusted for race, educational level, income, recreational activity, work activity, drinking status, smoking status, diabetes, and total energy intake (continuous) additionally. And we performed stratified analysis by gender to assess the association between dietary n3, n6 fatty acids intakes and n6:n3 ratio and the risk of hypertension. We further used restricted cubic spline with 3 knots located at the 5th, 50^th^, and 95th percentiles of the exposure distribution to assess dose-response relationship, and the adjusted confounding factors in restricted cubic spline were the same as those adjusted in the logistic regression model 2. Considering that the participants who had a history of hypertension might change their dietary pattern, we further excluded these participants and conducted binary logistic regression analyses to examine the association of dietary n3, n6 fatty acids intake and n6:n3 ratio with newly diagnosed hypertension and conducted multivariate logistic regression to examine the association of dietary n3, n6 fatty acids intake and n6:n3 ratio with stage 1 and stage 2 hypertension. We tested the value of the second zero spline coefficient to calculate the non-linear *p* value. In order to conduct a nationally representative estimate, analyses in this study were weighted, and the weights took into account the stratified, multistage probability sampling design and survey nonresponse. All statistical analyses were conducted by Stata 12.0 (Stata Corporation, College Station, TX, USA). A two-sided *p* <0.05 was considered statistically significant.

## 3. Results

The baseline characteristics of participants by hypertension are shown in Table 1. Finally, a total of 18,434 participants were included in this cross-sectional study, among them, 48.9% were males. The prevalence of hypertension was 48.7%. Compared with participants without hypertension, those with hypertension tended to be older, more likely to be male, obese, and diabetic, had lower educational level and household income, more likely to smoke at least 100 cigarettes in their life, and had less total energy intake. There were no differences between the two groups in marital status and drinking status. Moreover, those with hypertension had less n3, n6 fatty acids intake and a lower n6:n3 ratio.

The weighted odds ratios (ORs) with 95% confidence intervals (Cis) of hypertension based on tertiles of dietary n3, n6 fatty acids intakes and n6:n3 ratio are shown in Table 2. In binary logistic regression analyses, compared with the lowest tertile, the ORs with 95% CIs of hypertension for the highest tertile of n3, n6 fatty acids intake and n6:n3 ratio were 0.62 (0.56–0.69), 0.58 (0.53–0.64) and 0.82 (0.73-0.93), respectively. In model 1, after adjusting for age and gender, n3 and n6 fatty acids intakes were still inversely associated with the risk of hypertension, but a negative association between n6:n3 ratio and the risk of hypertension disappeared. Further adjusted race, educational level, income, recreational activity, work activity, drinking status, smoking status, diabetes, and total energy intake in model 2, the ORs with 95% CIs of hypertension were 0.58 (0.49–0.68), 0.53 (0.45–0.63), and 0.92 (0.80–1.06) for n3, n6 fatty acids intake and n6:n3 ratio, respectively.

The association between n3, n6 fatty acids intakes and n6:n3 ratio and the risk of hypertension in stratified analyses by gender are shown in Table 3. Among males, the ORs with 95% CIs of hypertension were 0.61 (0.48–0.79), and 0.49 (0.38–0.63) in model 2 for n3 and n6 fatty acids intake, respectively. And the negative associations of n3 and n6 fatty acids intakes and the risk of hypertension were also found in females. Among males, the association of n6:n3 ratio and the risk of hypertension was only significant in the unadjusted model, and was not significant in model 1 and model 2. The association of n6:n3 ratio and the risk of hypertension in females were similar to the results in males.

**Figure 2:**
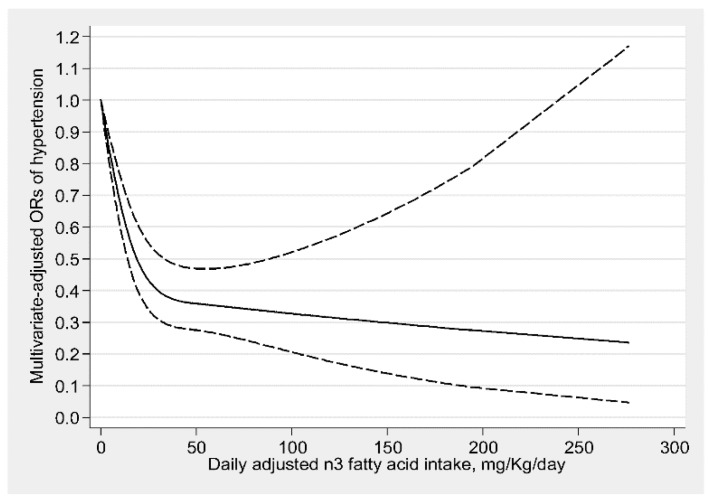
Dose-response relationship between n3 fatty acid intake and hypertension. The association was adjusted for age, gender, continuous race, educational level, income, recreational activity, work activity, drinking status, smoking status, diabetes, and total energy intake. The solid line and dash line represent the estimated ORs and its 95% confidence intervals. (OR, odds ratio).

**Figure 3:**
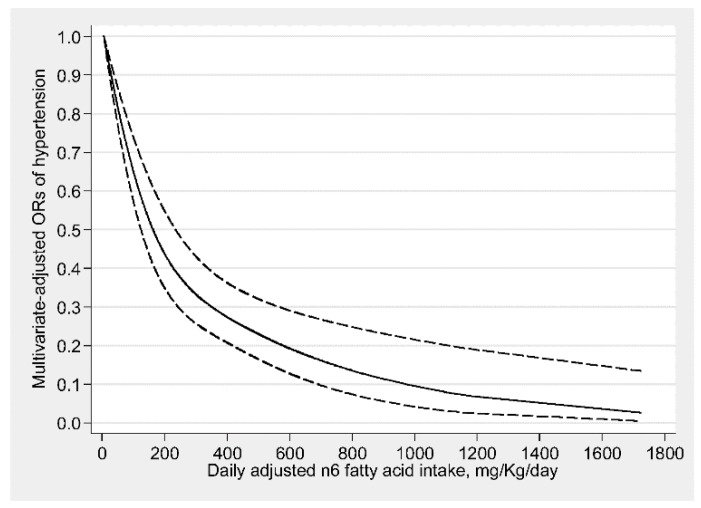
Dose-response relationship between n6 fatty acid intake and hypertension. The association was adjusted for age, gender, continuous race, educational level, income, recreational activity, work activity, drinking status, smoking status, diabetes, and total energy intake. The solid line and dash line represent the estimated ORs and its 95% confidence intervals. (OR, odds ratio).

The dose-response relationship between n3, n6 fatty acids intakes and the risk of hypertension are shown in Figure 2; Figure 3, respectively. There was a nonlinear negative and L-shaped association between n3 fatty acid intake and the risk of hypertension (*p* for nonlinearity <0.001), and there was no further significant reduction in hypertension risk beyond 45 mg/kg/day (OR: 0.36; 95% CI: 0.28–0.47). With an increase of n3 fatty acid intake, there was no significant association in hypertension risk beyond 240 mg/kg/day (OR: 0.26; 95% CI: 0.08–1.01) (Figure 2). There also existed a nonlinear negative and L-shaped association between n6 fatty acid intake and the risk of hypertension (*p* for nonlinearity <0.05), and the prevalence of hypertension decreased with increasing n6 fatty acid intake and reached a plateau when n6 fatty acid intake above 400 mg/kg/day (OR: 0.27; 95% CI: 0.21–0.36) (Figure 3). The adjusted confounding factors in restricted cubic spline were the same as those adjusted in the logistic regression model 2. 

Considering that the participants who had a history of hypertension might change their dietary pattern, we further excluded 6,488 participants who had hypertension history or were taking medications for hypertension. The weighted ORs with 95% CIs of newly diagnosed hypertension, stage 1 and stage 2 hypertension based on tertiles of dietary n3, n6 fatty acids intakes and n6:n3 ratio are shown in Table 4. There were significant negative correlations between n3, n6 fatty acids intakes and the risk of newly diagnosed hypertension. After newly diagnosed hypertension being further divided into stage 1 and stage 2 hypertension, the results did not change significantly and all the results were still stable.

## 4. Discussion

To our knowledge, this is the first study to explore the associations between n3, n6 fatty acids intakes and n6:n3 ratio and the risk of hypertension among the general American population after the 2017 new hypertension diagnostic criteria was released. Our study indicated that the intakes of n3 and n6 fatty acids were associated decreased risk of hypertension. At the same time, the analyses of different stage hypertension and dose-response relationship also found stable results.

The results of our study were consistent with some previous studies [17,35,36,37]. Levinson et al. [35] found n3 fatty acid intake could reduce the risk of hypertension, and the study conducted by Knapp et al. [36] also found this negative association. Meanwhile, our study also indicated n6 fatty acid intake could reduce the risk of hypertension, and the study conducted by Nakamura et al. [17] also indicated this negative association. The association between n6:n3 ratio and the risk of hypertension was not significant in our study, and Mirmiran et al. [37] also found this not significant association. Besides, some randomized controlled studies [43,44] indicated n3 fatty acid intake could reduce the level of blood pressure and study conducted by Skilton et al. [45] also found n3 fatty acid intake could reduce blood pressure. In contrast, Djousse et al. [39] found not statistically significant association between n6 fatty acid intake and the risk of hypertension. Mirmiran et al. [37] found there were no associations between n3, n6 fatty acids intake and the risk of hypertension among the Iranian population. Wang et al. [38] also found that these associations between n3, n6 fatty acids intake and the risk of hypertension among US women were not statistically significant.

N3 and N6 fatty acids belong to polyunsaturated fatty acids. Polyunsaturated fatty acids have the following physiological functions: (1) Esterification of cholesterol, reduce blood cholesterol and triglycerides; (2) reduce platelet aggregation, reduce thrombosis; (3) reduce blood viscosity, improve blood microcirculation and so on. At the same time, eicosatetraenoic acid (EPA) and docosahexaenoic acid (DHA), the two main n3 fatty acids, have multiple effects, such as improving arterial compliance [46], antiplatelet [47], anti-inflammatory, and reducing oxidative stress [48]. However, the mechanisms between n3, n6 fatty acid intake and the decreased hypertension risk are not fully understood, but several possible mechanisms have been suggested. First, n3 fatty acid could influence the synthesis of arachidonic acid and eicosanoids, thus producing an anti-inflammatory effect [49,50], and we all know inflammatory effects play an important role in the occurrence and development of hypertension. The anti-inflammatory effect could also explain the mechanism between n3 fatty acid and the decreased hypertension risk [51,52]. Second, n3 fatty acid could enhance endothelial vasodilator function [53], reduce reactivity of resistant vessel vascular smooth muscle [54], and increase vascular compliance [46]. Third, n3 fatty acid also could reduce angiotensin-converting enzyme activity [55], further inhibit renin-angiotensin-aldosterone system, and reduce the level of blood pressure. Fourth, in the molecular aspect, n3 and n6 fatty acids have the ability to regulate and they can act as signal molecules on peroxisome proliferator-activated receptors (PPARs), which are known to regulate several metabolic processes, including lipid and glucose metabolism, adipogenesis, inflammatory responses, or oxidative stress [56]. In addition, antioxidation [57], inhibition of thromboxane production, and reduction of plasma homocysteine level [58] could also be used to explain the mechanisms between n3, n6 fatty acid intake and the decreased hypertension risk.

There are several advantages in our study. First, we used 2017 new hypertension diagnostic criteria and conducted this cross-sectional study among the general American population. Second, we investigated the dose-response relationship between dietary n3 and n6 fatty acids intake and the risk of hypertension. Third, considering that the participants who had hypertension history might change their dietary pattern, we further excluded participants who had hypertension history or were taking medications for hypertension, and all results were still statistically significant. Fourth, the large sample size improved the statistical power and reliability of the results. 

However, there were also several limitations in our study. First, our study is a cross-sectional study, and it is difficult for us to determine causality. Second, we try to control the confounding factors as much as possible, but we cannot control the confounding factors completely. Third, there was not a detailed classification of linolenic acid in NHANES and we categorized linolenic acid as a n3 fatty acid, this might lead to the results not being very precise. Fourth, the dietary data were from two 24 h dietary recall interviews, this dietary survey method is often imprecise because of conscious or unconscious mis-recordings and under-reporting [59] and there might be ineluctable recall bias. In addition, we could not obtain the intake of n3 fatty acid and n6 fatty acid from dietary supplements, which was an important source of n3 fatty acid and n6 fatty acid.

## 5. Conclusions

In conclusion, our study suggests that dietary n3 and n6 fatty acids intake might be inversely associated with the risk of hypertension in US adults. Further large-scale prospective studies and studies with more accurate dietary survey methods are required to confirm these findings.

## Figures and Tables

**Table 1:** Characteristics of participants by hypertension, NHANES 2007–2014 (*N* = 18,434).

	Non-Hypertension	Hypertension	*p* Value
**Number of Participants (%)**	9458 (51.3)	8976 (48.7)	
**Age group (%) ^1^**			<0.01
18–39 years	5212 (55.1)	1245 (13.9)	
40–59 years	2956 (31.3)	3026 (33.7)	
60–79 years	1127 (11.9)	3783 (42.1)	
≥80 years	163 (1.7)	922 (10.3)	
**Gender (%) ^1^**			<0.01
Male	4403 (46.6)	4613 (51.4)	
Female	5055 (53.4)	4363 (48.6)	
**Race (%) ^1^**			<0.01
Mexican American	1670 (17.7)	1041 (11.6)	
Other Hispanic	1083 (11.5)	767 (8.5)	
Non-Hispanic White	4108 (43.4)	4254 (47.4)	
Non-Hispanic Black	1584 (16.7)	2302 (25.6)	
Other race	1013 (10.7)	612 (6.8)	
**Educational level (%) ^1^**			<0.01
Below high school	2197 (23.2)	2435 (27.2)	
High school	2061 (21.8)	2233 (24.9)	
Above high school	5192 (54.9)	4299 (27.9)	
**Marital status (%) ^1^**			0.706
Married/Living with partner	5141 (59.6)	5276 (59.4)	
Widowed/Divorced/Separated/Never married	3478 (40.4)	3611 (40.6)	
**Household income (%) ^1^**			<0.01
<$20,000	1770 (19.5)	2067 (23.9)	
≥$20,000	7293 (80.5)	6570 (76.1)	
**Body mass index (%) ^1^**			<0.01
<25 kg/m^2^	3660 (38.7)	1850 (20.7)	
25 to <30 kg/m^2^	3113 (32.9)	2917 (32.6)	
≥30 kg/m^2^	2677 (28.3)	4179 (46.7)	
**Work activity (%) ^1^**			<0.01
Vigorous activity	1871 (19.8)	1470 (16.4)	
Moderate activity	2118 (22.4)	1861 (20.7)	
Other	5469 (57.8)	5645 (62.9)	
**Recreational activity (%) ^1^**			<0.01
Vigorous activity	2815 (29.8)	1216 (13.5)	
Moderate activity	2414 (25.5)	2524 (28.1)	
Other	4229 (44.7)	5236 (58.3)	
**Smoking at least 100 cigarettes in life (%) ^1^**	3663 (38.7)	4287 (47.8)	<0.01
**Have at least 12 alcohol drinks/year (%) ^1^**	6351 (67.1)	6013 (67.0)	0.818
**Diabetes (%) ^1^**	446 (4.7)	1757 (19.6)	<0.01
**Total energy intake (kcal/day) ^2^**	1977 (1043)	1837.5 (957.5)	<0.01
**Total adjusted n3 fatty acid intake (mg/kg/day) ^2^**	20.37 (16.05)	17.51 (13.92)	<0.01
**Total adjusted n6 fatty acid intake (mg/kg/day) ^2^**	189.16 (141.81)	159.24 (121.98)	<0.01
**n6:n3 ratio ^2^**	9.11 (3.17)	8.91 (3.05)	<0.01

Data are the number of subjects (percentage) or medians (interquartile ranges). ^1^ Chi-square test was used to compare the percentage between participants with and without hypertension. ^2^ Mann-Whitney *U* test was used to compare the mean values between participants with and without hypertension. National Health and Nutrition Examination Survey (NHANES).

**Table 2:** Weighted odds ratios (95% confidence intervals) of hypertension across tertiles of adjusted dietary n3, n6 fatty acids intake and n6:n3 ratio, NHANES 2007–2014 (*N* = 18,434).

	Case/Participants	Crude ^1^	Model 1 ^1^	Model 2 ^1^
**Adjusted n3 (mg/Kg/day)**				
<14.65	3398/6138	1.00 (Ref.)	1.00 (Ref.)	1.00 (Ref.)
14.65 to <24.32	3036/6158	0.79 (0.71–0.88) **	0.78 (0.68–0.90) **	0.78 (0.67–0.91) **
≥24.32	2542/6138	0.62 (0.56–0.69) **	0.58 (0.52–0.65) **	0.58 (0.49–0.68) **
**Adjusted n6 (mg/Kg/day)**				
<135.78	3460/6138	1.00 (Ref.)	1.00 (Ref.)	1.00 (Ref.)
135.78 to <221.11	3029/6158	0.75 (0.68–0.83) **	0.71 (0.62–0.80) **	0.70 (0.62–0.80) **
≥221.11	2487/6138	0.58 (0.53–0.64) **	0.58 (0.52–0.65) **	0.53 (0.45–0.63) **
**n6:n3 ratio**				
<8.11	3143/6138	1.00 (Ref.)	1.00 (Ref.)	1.00 (Ref.)
8.11 to <10.04	3023/6158	1.00 (0.91–1.10)	1.13 (1.01–1.26) *	1.10 (0.99–1.23)
≥10.04	2810/6138	0.82 (0.73–0.93) **	0.96 (0.84–1.09)	0.92 (0.80–1.06)

^1^ Calculated using binary logistic regression. Model 1 adjusted for age and gender. Model 2 adjusted for age and gender, race, educational level, income, recreational activity, work activity, drinking status, smoking status, diabetes, and total energy intake. * *p* <0.05; ** *p* <0.01.

**Table 3:** Weighted odds ratios (95% confidence intervals) of hypertension across tertiles of adjusted dietary n3, n6 fatty acid intake and n6:n3 ratio, stratified by gender, NHANES 2007–2014 (*N* = 18,434).

	Male			Female		
	Crude ^1^	Model 1 ^1^	Model ^1^	Crude ^1^	Model 1 ^1^	Model 2 ^1^
**Adjusted n3 (mg/Kg/day)**						
<14.65	1.00 (Ref.)	1.00 (Ref.)	1.00 (Ref.)	1.00 (Ref.)	1.00 (Ref.)	1.00 (Ref.)
14.65 to <24.32	0.74 (0.64–0.87) **	0.77 (0.64–0.93) **	0.77 (0.63–0.95) *	0.81 (0.69–0.96) *	0.78 (0.64–0.95) *	0.78 (0.63–0.97) *
≥24.32	0.60 (0.51–0.71) **	0.61 (0.51–0.73) **	0.61 (0.48–0.79) **	0.62 (0.53–0.72) **	0.54 (0.46–0.65) **	0.53 (0.41–0.68) **
**Adjusted n6 (mg/Kg/day)**						
<135.78	1.00 (Ref.)	1.00 (Ref.)	1.00 (Ref.)	1.00 (Ref.)	1.00 (Ref.)	1.00 (Ref.)
135.78 to <221.11	0.66 (0.56–0.77) **	0.65 (0.54–0.78) **	0.63 (0.52–0.75) **	0.81 (0.68–0.96) *	0.76 (0.62–0.92) **	0.76 (0.61–0.96) *
≥221.11	0.53 (0.46–0.61) **	0.56 (0.47–0.66) **	0.49 (0.38–0.63) **	0.60 (0.51–0.70) **	0.59 (0.49–0.70) **	0.56 (0.43–0.72) **
**n6:n3 ratio**						
<8.11	1.00 (Ref.)	1.00 (Ref.)	1.00 (Ref.)	1.00 (Ref.)	1.00 (Ref.)	1.00 (Ref.)
8.11 to <10.04	0.94 (0.81–1.08)	1.07 (0.91–1.25)	1.05 (0.90–1.24)	1.06 (0.91–1.23)	1.18 (0.98–1.42)	1.14 (0.95–1.38)
≥10.04	0.80 (0.68–0.95) *	0.90 (0.76–1.08)	0.87 (0.73–1.05)	0.83 (0.70–0.97) *	1.02 (0.84–1.24)	0.98 (0.81–1.20)

^1^ Calculated using binary logistic regression. Model 1 adjusted for age. Model 2 adjusted for age, race, educational level, income, recreational activity, work activity, drinking status, smoking status, diabetes, and total energy intake. * *p* < 0.05; ** *p* < 0.01.

**Table 4:** Weighted odds ratios (95% confidence intervals) of newly diagnosed hypertension across tertiles of adjusted dietary n3, n6 fatty acids intake and n6:n3 ratio, NHANES 2007–2014 (*N* = 11,946).

	Hypertension			Stage 1 Hypertension			Stage 2 Hypertension		
	Crude ^1^	Model 1 ^1^	Model 2 ^1^	Crude ^2^	Model 1 ^2^	Model 2 ^2^	Crude ^2^	Model 1 ^2^	Model 2 ^2^
**Adjusted n3 (mg/Kg/day)**									
<15.46	1.00 (Ref.)	1.00 (Ref.)	1.00 (Ref.)	1.00 (Ref.)	1.00 (Ref.)	1.00 (Ref.)	1.00 (Ref.)	1.00 (Ref.)	1.00 (Ref.)
15.46 to <25.58	0.82 (0.71–0.96) *	0.81 (0.69–0.96) *	0.76 (0.64–0.91) **	0.89 (0.80–1.01)	0.86 (0.77–0.97) *	0.82 (0.72–0.93) **	0.86 (0.74–0.99) *	0.86 (0.74–1.01)	0.82 (0.69–0.96) *
≥25.58	0.71 (0.62–0.80) **	0.67 (0.59–0.77) **	0.60 (0.50–0.73) **	0.73 (0.64–0.82) **	0.70 (0.62–0.80) **	0.63 (0.54–0.74) **	0.64 (0.55–0.75) **	0.66 (0.56–0.77) **	0.63 (0.51–0.77) **
**Adjusted n6 (mg/Kg/day)**									
<144.81	1.00 (Ref.)	1.00 (Ref.)	1.00 (Ref.)	1.00 (Ref.)	1.00 (Ref.)	1.00 (Ref.)	1.00 (Ref.)	1.00 (Ref.)	1.00 (Ref.)
144.81 to <233.31	0.79 (0.69–0.90) **	0.75 (0.65–0.86) **	0.69 (0.59–0.80) **	0.85 (0.75–0.95) **	0.82 (0.73–0.93) **	0.77 (0.68–0.88) **	0.69 (0.60–0.80) **	0.71 (0.61–0.83) **	0.66 (0.56–0.78) **
≥233.31	0.65 (0.57–0.73) **	0.63 (0.55–0.71) **	0.52 (0.43–0.62) **	0.70 (0.62–0.78) **	0.70 (0.62–0.79) **	0.57 (0.49–0.67) **	0.54 (0.46–0.63) **	0.62 (0.52–0.73) **	0.54 (0.44–0.67) **
**n6:n3 ratio**									
<8.17	1.00 (Ref.)	1.00 (Ref.)	1.00 (Ref.)	1.00 (Ref.)	1.00 (Ref.)	1.00 (Ref.)	1.00 (Ref.)	1.00 (Ref.)	1.00 (Ref.)
8.17 to <10.13	1.07 (0.93–1.23)	1.16 (1.00–1.35)	1.15 (0.99–1.33)	0.98 (0.87–1.10)	1.06 (0.94–1.19)	1.04 (0.92–1.18)	0.86 (0.75–1.00)	1.03 (0.88–1.21)	0.99 (0.84–1.17)
≥10.13	0.90 (0.77–1.06)	0.97 (0.81–1.14)	0.95 (0.79–1.14)	0.93 (0.83–1.05)	1.00 (0.89–1.14)	0.96 (0.85–1.09)	0.73 (0.63–0.85) **	0.91 (0.78–1.08)	0.85 (0.72–1.01)

^1^ Calculated using binary logistic regression. ^2^ Calculated using multinomial logistic regression. Model 1 adjusted for age and gender. Model 2 adjusted for age and gender, race, educational level, income, recreational activity, work activity, drinking status, smoking status, diabetes, and total energy intake. * *p* < 0.05; ** *p* < 0.01.
